# Author Correction: The endocannabinoid N-arachidonoyl dopamine is critical for hyperalgesia induced by chronic sleep disruption

**DOI:** 10.1038/s41467-023-43155-9

**Published:** 2023-11-13

**Authors:** Weihua Ding, Liuyue Yang, Eleanor Shi, Bowon Kim, Sarah Low, Kun Hu, Lei Gao, Ping Chen, Wei Ding, David Borsook, Andrew Luo, Jee Hyun Choi, Changning Wang, Oluwaseun Akeju, Jun Yang, Chongzhao Ran, Kristin L. Schreiber, Jianren Mao, Qian Chen, Guoping Feng, Shiqian Shen

**Affiliations:** 1 Department of Anesthesia, Critical Care and Pain Medicine, Massachusetts General Hospital, Harvard Medical School, Boston, MA USA; 2 Department of Pathology, Tuft University School of Medicine, Boston, MA USA; 3 College of Science and Mathematics, University of Massachusetts Boston, Boston, MA USA; 4 Department of Radiology, Massachusetts General Hospital, Harvard Medical School, Boston, MA USA; 5 Summer Intern Program of the Department of Anesthesia, Critical Care and Pain Medicine, Massachusetts General Hospital, currently at Brandeis University, Boston, MA USA; 6 Center for Neuroscience, Korea Institute of Science and Technology, Seoul, South Korea; 7 Martinos Center for Biomedical Imaging, Department of Radiology, Massachusetts General Hospital, Harvard Medical School, Boston, MA USA; 8 Department of Anesthesiology, Perioperative and Pain Medicine, Brigham and Women’s Hospital, Harvard Medical School, Boston, MA USA; 9 McGovern Institute for Brain Research and Department of Brain and Cognitive Sciences, Massachusetts Institute of Technology, Cambridge, MA USA; 10 Present Address: Zhongshan Institute for Drug Discovery, Shanghai Institute of Materia Medica, Chinese Academy of Sciences, Shanghai, China

**Keywords:** Chronic pain, Sleep

Correction to: *Nature Communications* 10.1038/s41467-023-42283-6, published online 25 October 2023

In this article, the affiliation details for Guoping Feng were incorrectly given as ‘Zhongshan Institute for Drug Discovery, Shanghai Institute of Materia Medica, Chinese Academy of Sciences, Shanghai, China’ but should have been ‘McGovern Institute for Brain Research and Department of Brain and Cognitive Sciences, Massachusetts Institute of Technology, Cambridge, MA, USA’. The original article has been corrected.

