# Supplementary material for: The endocannabinoid N-arachidonoyl dopamine is critical for hyperalgesia induced by chronic sleep disruption
Source: Nat Commun. 2023 Oct 25;14:6696. doi: 10.1038/s41467-023-42283-6 (PMC10600211; doi:10.1038/s41467-023-42283-6)
Supplement: Supplementary file 1 — Supplementary Information [file 41467_2023_42283_MOESM1_ESM.pdf]

The endocannabinoid N-arachidonoyl dopamine is critical for hyperalgesia induced by chronic sleep disruption

Weihua Ding[1*], Liuyue Yang[1*], Eleanor Shi[1], Bowon Kim[1], Sarah Low[1], Kun Hu[2], Lei Gao[1], Ping Chen[3], Wei Ding[3], David Borsook[4], Andrew Luo[5], Jee Hyun Choi[6], Changning Wang[7], Oluwaseun Akeju[1], Jun Yang[7], Chongzhao Ran[7], Kristin L. Schreiber[8], Jianren Mao[1], Qian Chen[9,10], Guoping Feng[10†], Shiqian Shen[1†]

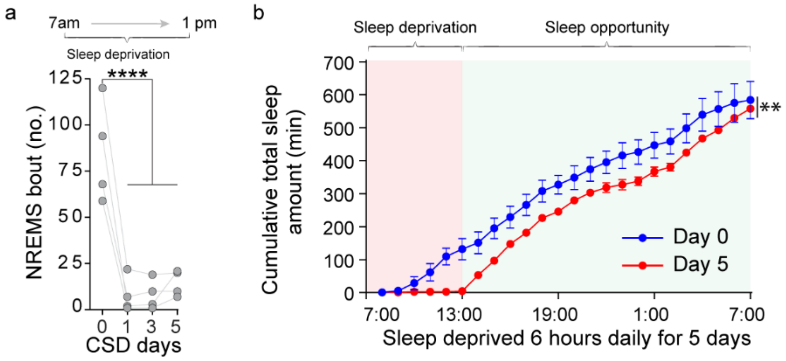

Supplementary Figure 1. Chronic sleep disruption (CSD)

a-b Quantification of sleep during CSD. Four mice were subjected to wireless EEG. **a** Two-hour EEG (1-3 pm) was performed immediately after CSD sessions. Bouts of nonrapid eye movement sleep (NREM) were examined during CSD (n = 4 mice). One-way ANOVA was used for statistical analysis. ****p<0.0001. **b** Total sleep time during a 24-hour period on day 0 and day 5. Two-way ANOVA indicated that the difference between day 5 and day 0 was significant; data are presented as mean ± SEM, **p<0.01. Source data are provided as a Source Data file.

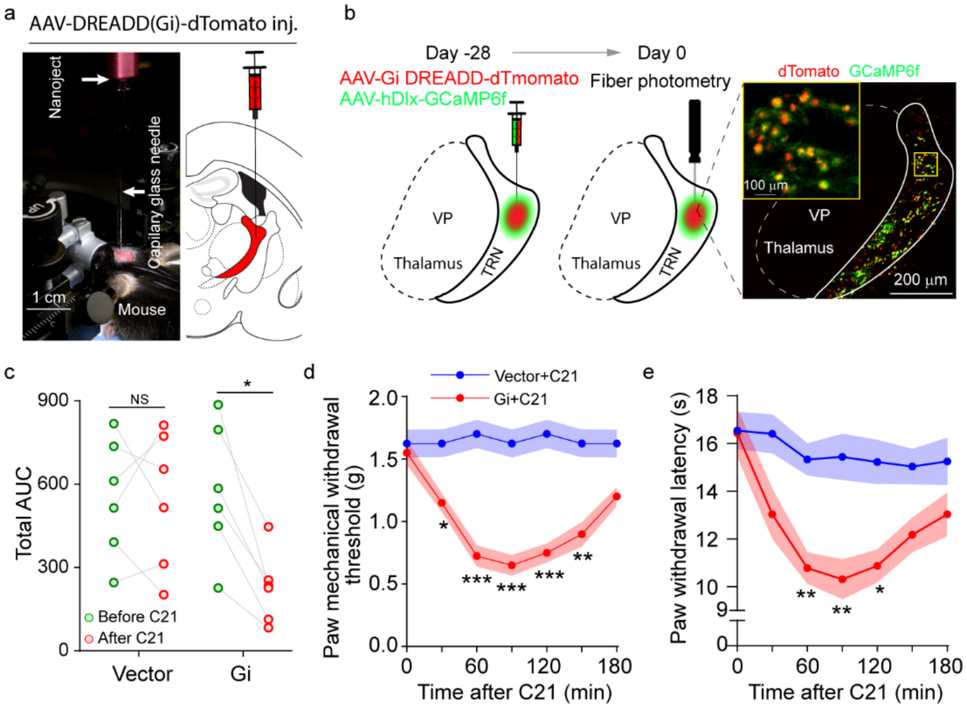

Supplementary Figure 2. The TRN is critical for CSD-induced hyperalgesia. a Image of the AAV injection setup and diagram of AAV injection into the TRN. **b and c** Chemogenetic inhibition of TRN. Vector AAV-hDlx-dTomato control or AAV-Dlx-Gi DREADDs-dTomato was injected together with AAV-Dlx-GCaMP6f to the TRN (n = 6 mice). **b** Flowchart and sketch depict experiment design. A representative image of virus expression in TRN. **c** Total area under the curve (AUC) was calculated in Vector control or Gi DREADDs group, before and after C21 administration (n = 6 mice). Two-tailed paired t test, *$p<0.05$. **d and e** Chemogenetic inhibition of the TRN promoted nociceptive behavior (n = 8 mice). Targeted inhibition of TRN activity using AAV-hDlx-Gi GREADD-dTomato or AAV-hDlx-dTomato as a vector control. Hindpaw mechanical withdrawal threshold **d** and hindpaw thermal withdrawal latency **e** were examined at the indicated time points. Two-way ANOVA indicated that the differences in behavioral parameters between the two groups were statistically significant. The Bonferroni post hoc test indicated that the differences were statistically significant at the indicated time points; data are presented as mean ± SEM, *$p<0.05$, **$p<0.01$, ***$p<0.001$. Source data are provided as a Source Data file.

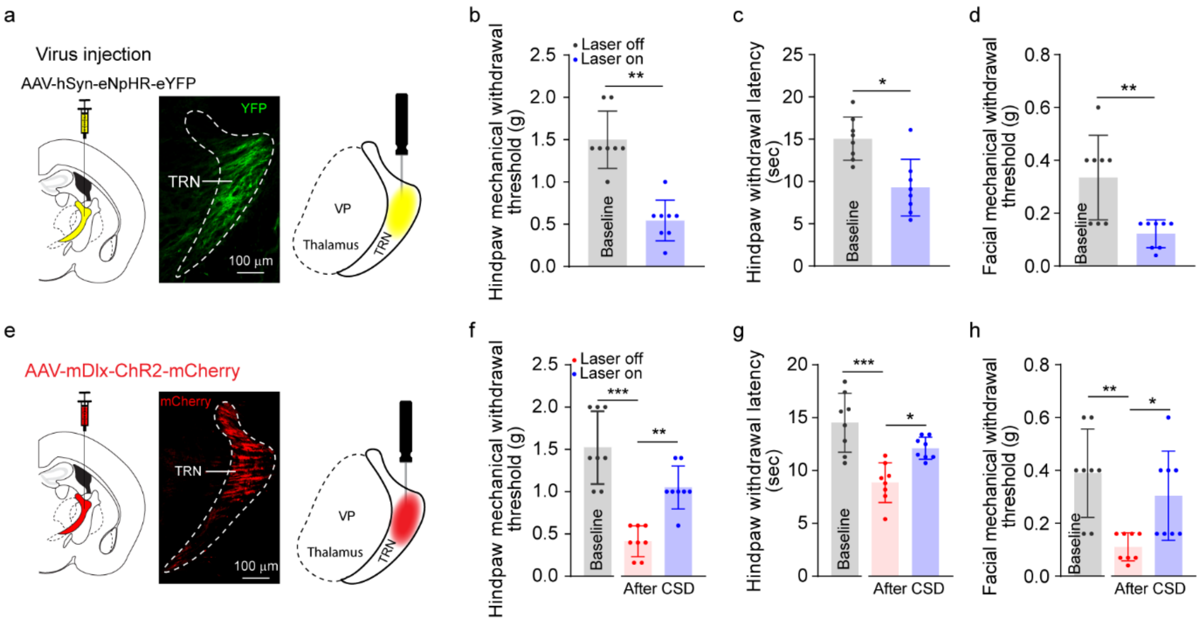

Supplementary Figure 3. Optogenetic manipulation of the TRN modulates pain-like behavior. a-d Optogenetic inhibition of the TRN led to hyperalgesia. **a** AAV-hSyn-eNpHR-EYFP was injected into the TRN followed by optical fiber implantation. A representative image of brain slice was shown to demonstrate virus expression in the TRN. Well-rested 'naïve' mice (*n* = 8 mice) were tested for behavior with laser off and laser on. **b** Hindpaw mechanical withdrawal threshold, **c** hindpaw withdrawal latency, and **d** facial mechanical withdrawal (*n* = 8 mice). Data were presented as mean ± SD. Two-tailed paired t test was used for comparison. **p*<0.05, ***p*<0.01. **e-h** Optogenetic activation of TRN dampens CSD-induced hyperalgesia. Mice underwent TRN injection and fiber implantation followed by CSD sessions. Behavior test was performed before CSD sessions, and after CSD sessions (laser off and on). **e** Sketch depicts virus injection into TRN with a representative brain slice imaging showing virus expression in the TRN. Behavior test includes **f** hindpaw mechanical withdrawal threshold, **g** hindpaw withdrawal latency and **h** facial mechanical withdrawal threshold was performed (*n* = 8 mice). Data are presented as mean ± SD. One-way ANOVA followed by Tukey post hoc test, **p*<0.05, ***p*<0.01, ****p*<0.001. Source data are provided as a Source Data file.

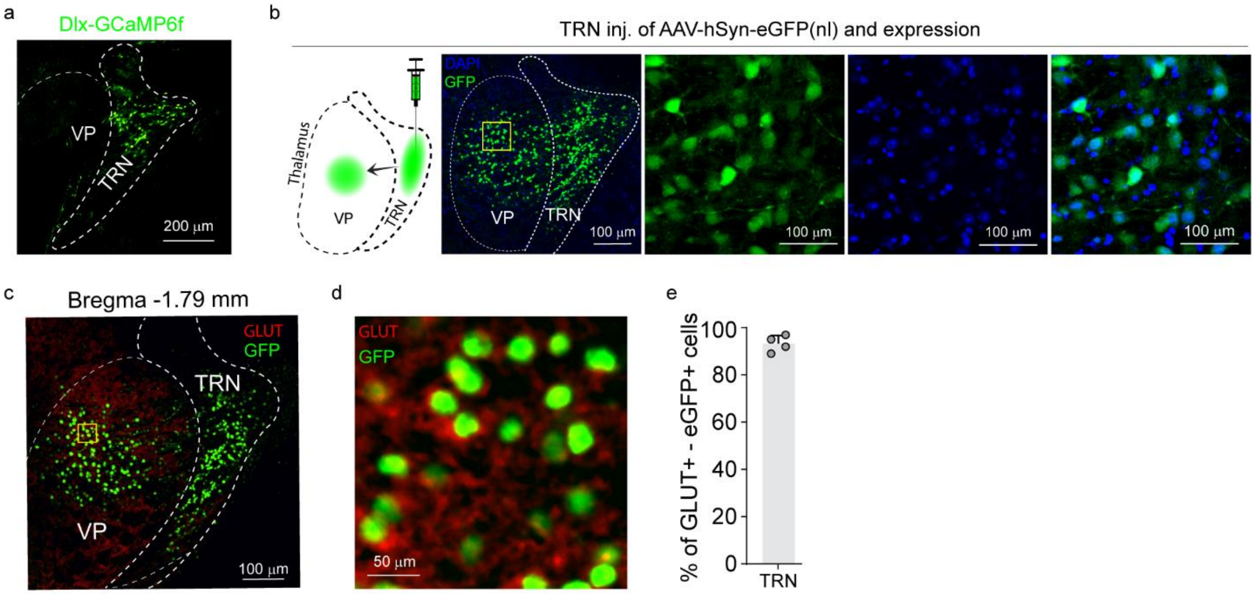

Supplementary Figure 4. The TRN to VP projection is implicated in CSD-induced hyperalgesia. a A representative picture of the expression of AAV-hDlx-GCaMP6f in the TRN (from 6 mice). **b-e** TRN projections to the VP. **b** AAV1-hSyn-eGFP-nls (nuclear localization) was injected into the TRN. The fluorescence image is representative of 4 independent injections, green: GFP, blue: DAPI. **c and d** The virus was significantly expressed in the VP. Red: glutamate, green: GFP. **e** The percentage of GFP-positive cells in the VP that were also positive for glutamate. VP: The ventral posterolateral nucleus; GLUT: glutamate; VP: The ventral posterolateral nucleus; GLUT: glutamate.

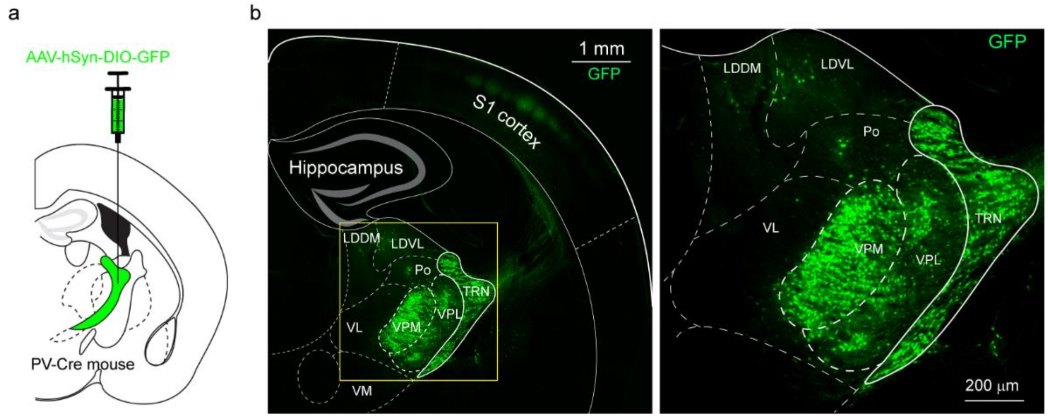

Supplementary Figure 5. Robust projection from TRN to VP in PV-Cre mouse. **a** Sketch depicts Cre-dependent virus AAV1-Syn-DIO-GFP injection into the TRN of PV-Cre mice (*n* = 3 mice). **b** Representative image with boxed region zoomed in showed strong GFP signals expressing in both TRN and VP along with light signals in Po, LDVL and LDDM of PV-Cre mouse. PV: parvalbumin; VPM: ventral posteromedial nucleus; VPL: ventral posterolateral nucleus; LDDM: laterodorsal dorsomedial thalamus; LDVL: laterodorsal ventrolateral part; Po: posterior thalamic nucleus; VL: ventral lateral nucleus.

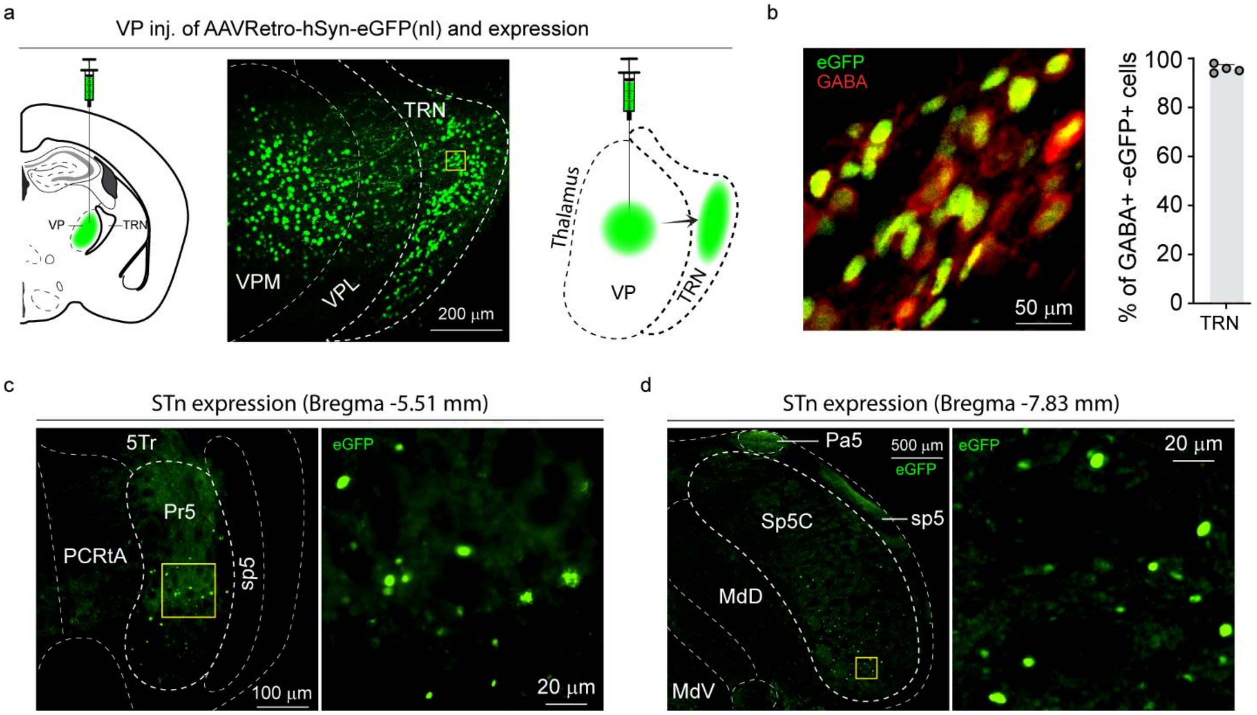

Supplementary Figure 6. AAVretro tracing. a Injection of AAVretro-hSyn-eGFP-nls (nuclear localization) into the VP. The fluorescence image is representative of 4 mice. **b** The TRN was stained for GABA. Green: GFP encoded by the AAV-retro originating in the VP; red: GABA. The percentage of eGFP-expressing cells in the TRN that were also positive for GABA. **c and d** Injection of AAVretro-hSyn-eGFP-Cre into the VP led to GFP expression in the spinal trigeminal nucleus. Shown pictures represent 4 independent injections. **c** Pr5 nucleus. **d** Sp5C nucleus. Source data are provided as a Source Data file. Pr5: principal sensory nucleus of the trigeminal nerve; Sp5C: spinal trigeminal nucleus caudalis.

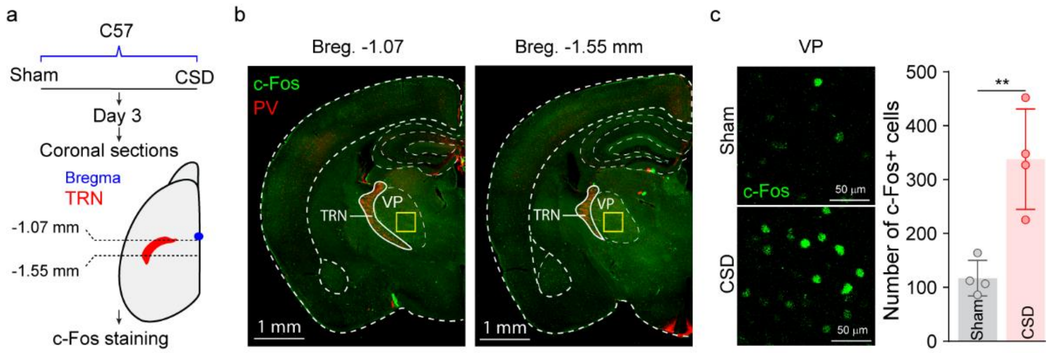

Supplementary Figure 7. CSD induces c-Fos expression. a Flowchart and sketch depict experiment design. **b** brain slices between bregma -1.07 and -1.55 mm were collected and co-stained with anti-c-Fos and anti-PV antibodies. VP region was used for statistics with boxed region shown in (c). **c** Representative images of c-Fos expression in VP, c-Fos$^+$ cells significantly increased in CSD mice compared to sham mice (n = 4 mice). Data are presented as mean ± SD. Two-tailed unpaired t test, **p<0.01. Source data are provided as a Source Data file.

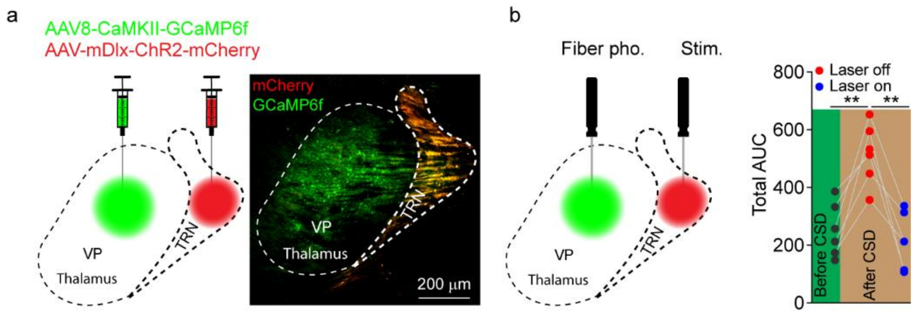

Supplementary Figure 8. Optogenetic activation of TRN alleviates CSD-induced VP hyperactivity. a Sketch depicts viruses injection into the TRN (AAV-Dlx-ChR2-mCherry) and VP (AAV-CaMKII-GCaMP6f). A representative image of brain slice was shown to demonstrate viral expression. Following TRN and VP injection, mice underwent CSD sessions, fiber photometry was carried out before CSD and after CSD sessions (laser off and on). **b** Simultaneous optogenetic stimulation of the TRN and fiber photometry of the VP were carried out. Total AUC (area under the curve) of VP calcium dynamics were plotted (n = 6 mice). One-way ANOVA followed by Tukey post hoc test was used for comparison. **p<0.01. Source data are provided as a Source Data file.

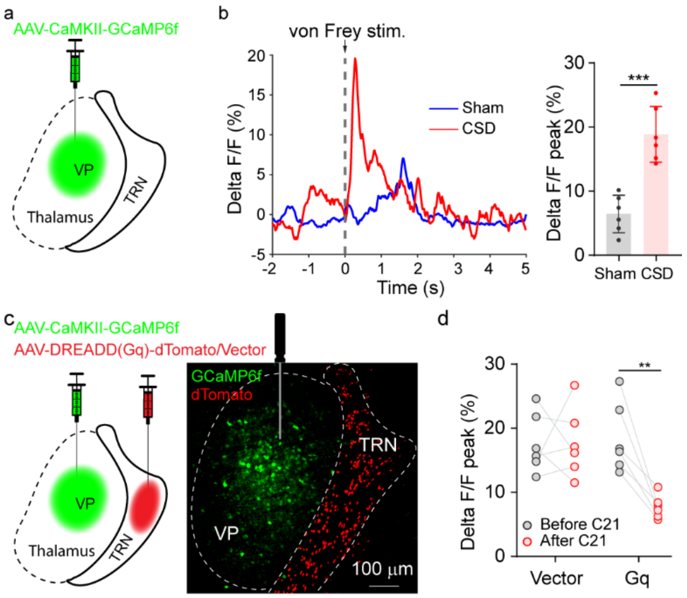

Supplementary Figure 9. CSD induces heightened VP activities in response to mechanical stimulation. **a-b** Mice underwent VP injection of AAV-CaMKII-GCaMP6f and fiber cannula implantation. After Sham or CSD sessions (*n* = 6 mice), facial area was stimulated with von Frey at 0.16 gram. **a** Sketch depicts virus injection into VP. **b** VP calcium dynamics in responses to von Frey stimulation. Left panel: sample traces; right panel: comparison between the Sham and CSD groups. ***p<0.001, two-tailed t test. **c-d** Mice underwent TRN injection of AAV-Dlx-Gq DREADDs-dTomato or vector control (*n* = 6 mice) along with VP injection of AAV-CaMKII-GCaMP6f. Optic fiber cannula was placed in the VP for fiber photometry. Mice underwent CSD sessions followed by VP calcium imaging in responses to von Frey at 0.16 gram, before and after administration of C21. **c** Sketch depicts virus injection into the VP and TRN, with a representative image of brain slice showing viruses expression in the VP and TRN. **d** Delta F/F peak in responses to facial von Frey at 0.16gram. Data are presented as mean ± SD, two-tailed unpaired t test, **p<0.01. Source data are provided as a Source Data file.

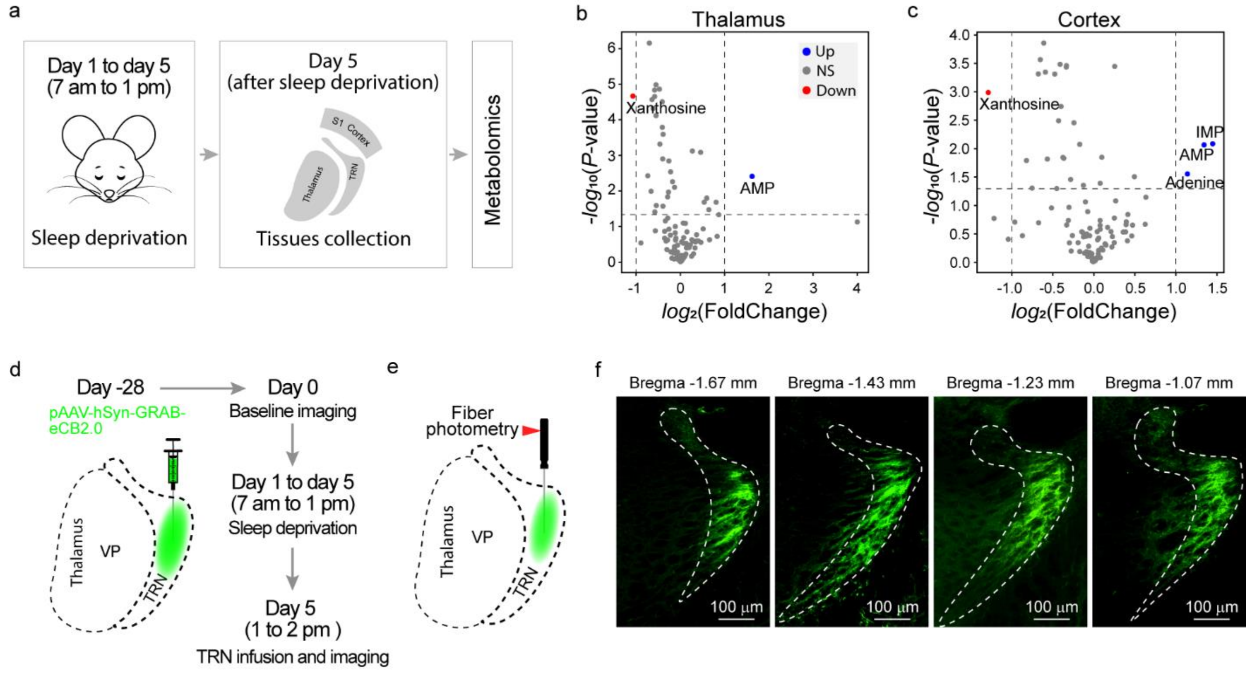

Supplementary Figure 10. NADA is decreased in the TRN after CSD. a-c Metabolomics study. **a** Diagram of the metabolomics study. **b-c** Volcano plots of metabolites in the thalamus (CSD *n* = 7 mice; control *n* = 8 mice). **b** and the primary somatosensory cortex (CSD *n* = 8 mice; *n* = 8 mice). **c** A fold change of 2 and p<0.05 (t test) were used as cutoffs. **d-f** CB1 receptor activity after CSD. AAV-hSyn-GRAB-eCB2.0 was injected into the TRN of mice (*n* = 6 mice), and the mice were implanted with fiber cannula. Four weeks were allowed for virus expression. **d, e** Diagram of the experimental design and fiber insertion site. **f** Tangential slices of the TRN stained with an anti-GFP antibody. The pictures are representative of a group of 6 animals.

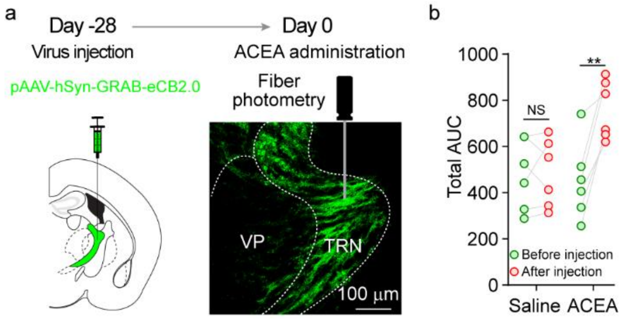

Supplementary Figure 11. CB1 receptor agonist Arachidonyl-2'-chloroethylamide (ACEA) increases GRAB-eCB2.0 activities. a Flowchart and sketch depict experimental design. A representative image of brain slice was shown to demonstrate GRAB-eCB2.0 expression and fiber photometry. **b** Total AUC (area under the curve) of GRAB-eCB2.0 of saline and ACEA treatment (n = 6 mice), two-tailed paired t test, **p<0.01; NS: p>0.05. Source data are provided as a Source Data file.

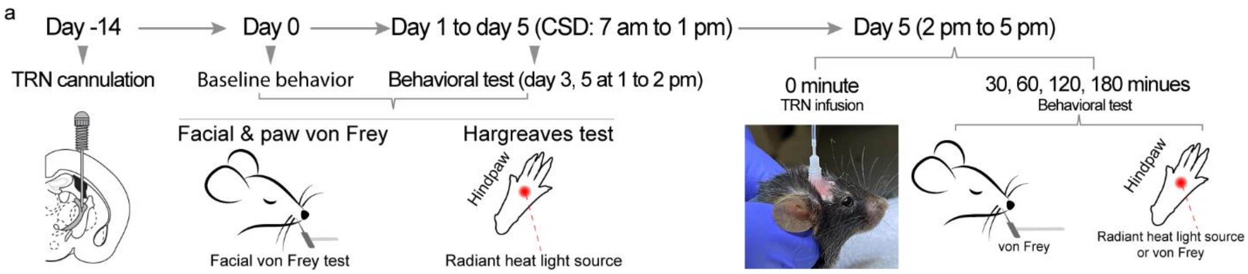

Supplementary Figure 12. a Diagram of the administration of NADA into the TRN and behavioral testing. NADA: N-Arachidonoyl dopamine.

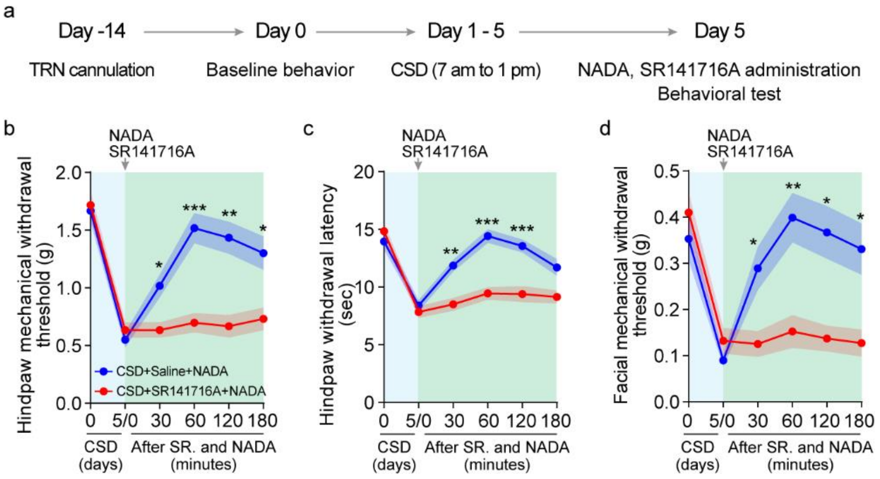

Supplementary Figure 13. CB1 receptor antagonist SR141716A abolishes NADA's effect on CSD-induced hyperalgesia. a Flowchart of experimental design. **b-d** The relief of CSD-induced hyperalgesia by NADA was abrogated by SR141716A. **b** Hindpaw mechanical withdrawal threshold, **c** hindpaw withdrawal latency, and **d** facial mechanical withdrawal threshold (n = 8 mice). Data presented as mean ± SEM. Two-way ANOVA followed by Bonferroni post hoc test was carried out for comparison between Saline vs. SR141716A group in NADA treated CSD mice, *p<0.05, **p<0.01, ***p<0.001. Source data are provided as a Source Data file.

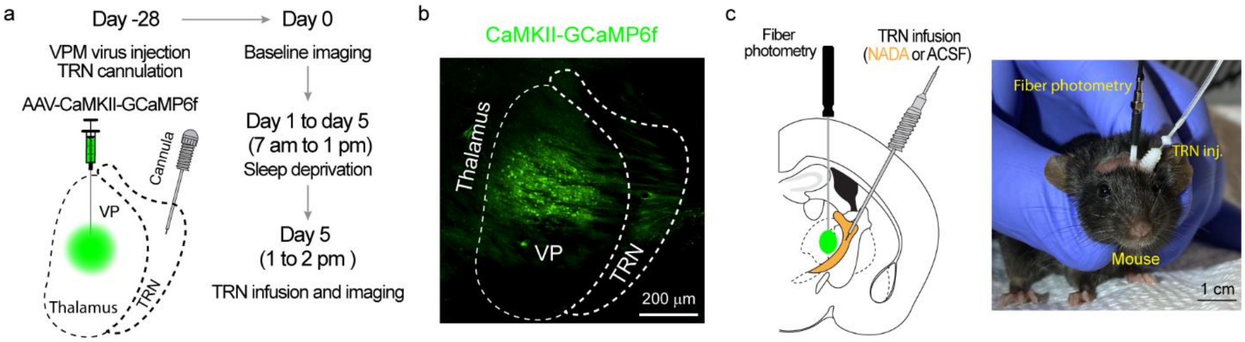

Supplementary Figure 14. Administration of NADA to the TRN dampened CSD-induced VP hyperactivity. The TRN was cannulated for NADA administration, and AAV-CaMKII-GCaMP6f was injected into the VP. Fiber photometry was performed on animals that received ACSF or NADA (*n* = 6 mice). **a** Diagram of TRN cannulation and fiber photometry of the VP. **b** Representative picture of virus expression in the VP. **c** Diagram and picture of NADA infusion with fiber photometry setup.
