# Supplementary material for: The endocannabinoid N-arachidonoyl dopamine is critical for hyperalgesia induced by chronic sleep disruption
Source: Nat Commun. 2023 Oct 25;14:6696. doi: 10.1038/s41467-023-42283-6 (PMC10600211; doi:10.1038/s41467-023-42283-6)
Supplement: Supplementary file 2 — Description of Additional Supplementary Files [file 41467_2023_42283_MOESM2_ESM.pdf]

Description of Additional Supplementary Files

File Name: Supplementary Data 1
Description: Mice were allocated into sham and CSD group (n=8 mice). At the end of the last session of sleep deprivation all mice were immediately sacrificed by decapitating using a disposable plastic cone with a guillotine. The cortex, thalamus and thalamic reticular nucleus were harvested respectively. A total of ~140 metabolites were measured in samples using LC-MS metabolite profiling techniques as described in PMID 37185230. In the supplementary data 1 file, measured metabolites were listed in the columns, and the samples were listed in rows.
