# Supplementary material for: The endocannabinoid N-arachidonoyl dopamine is critical for hyperalgesia induced by chronic sleep disruption
Source: Nat Commun. 2023 Oct 25;14:6696. doi: 10.1038/s41467-023-42283-6 (PMC10600211; doi:10.1038/s41467-023-42283-6)
Supplement: Supplementary file 4 — Reporting Summary [file 41467_2023_42283_MOESM4_ESM.pdf]

nature portfolio

Corresponding author(s): Dr. Shen, Shiqian; Guoping Feng

Last updated by author(s): Sep 6, 2023

Reporting Summary

Nature Portfolio wishes to improve the reproducibility of the work that we publish. This form provides structure for consistency and transparency in reporting. For further information on Nature Portfolio policies, see our Editorial Policies and the Editorial Policy Checklist.

Statistics

For all statistical analyses, confirm that the following items are present in the figure legend, table legend, main text, or Methods section.

n/a	Confirmed	
☐	☒	The exact sample size (*n*) for each experimental group/condition, given as a discrete number and unit of measurement
☐	☒	A statement on whether measurements were taken from distinct samples or whether the same sample was measured repeatedly
☐	☒	The statistical test(s) used AND whether they are one- or two-sided<br>*Only common tests should be described solely by name; describe more complex techniques in the Methods section.*
☒	☐	A description of all covariates tested
☐	☒	A description of any assumptions or corrections, such as tests of normality and adjustment for multiple comparisons
☐	☒	A full description of the statistical parameters including central tendency (e.g. means) or other basic estimates (e.g. regression coefficient) AND variation (e.g. standard deviation) or associated estimates of uncertainty (e.g. confidence intervals)
☐	☒	For null hypothesis testing, the test statistic (e.g. *F*, *t*, *r*) with confidence intervals, effect sizes, degrees of freedom and *P* value noted<br>*Give P values as exact values whenever suitable.*
☒	☐	For Bayesian analysis, information on the choice of priors and Markov chain Monte Carlo settings
☒	☐	For hierarchical and complex designs, identification of the appropriate level for tests and full reporting of outcomes
☐	☒	Estimates of effect sizes (e.g. Cohen's *d*, Pearson's *r*), indicating how they were calculated

Our web collection on statistics for biologists contains articles on many of the points above.

Software and code

Policy information about availability of computer code

Data collection	Immunofluorescence imaging using NIS-Elements (Nikon, Japan) imaging software and cellsens dimension (V4.2, Olympus, Japan) software; Matlab 2018b was used for fiber photometry and EEG/EMG recording
Data analysis	Matlab 2018b; ImageJ (NIH, V1.51); Graphpad Prism 8.0; Microsoft Excell

For manuscripts utilizing custom algorithms or software that are central to the research but not yet described in published literature, software must be made available to editors and reviewers. We strongly encourage code deposition in a community repository (e.g. GitHub). See the Nature Portfolio guidelines for submitting code & software for further information.

Data

Policy information about availability of data

All manuscripts must include a data availability statement. This statement should provide the following information, where applicable:

- Accession codes, unique identifiers, or web links for publicly available datasets
- A description of any restrictions on data availability
- For clinical datasets or third party data, please ensure that the statement adheres to our policy

Source data are provided with this paper. Construct of AAV-hSyn-GFP (nls) will be deposited to Addgene for access. Original imaging is available upon request.

Research involving human participants, their data, or biological material

Policy information about studies with human participants or human data. See also policy information about sex, gender (identity/presentation), and sexual orientation and race, ethnicity and racism.

Reporting on sex and gender	N/A
Reporting on race, ethnicity, or other socially relevant groupings	N/A
Population characteristics	N/A
Recruitment	N/A
Ethics oversight	N/A

Note that full information on the approval of the study protocol must also be provided in the manuscript.

Field-specific reporting

Please select the one below that is the best fit for your research. If you are not sure, read the appropriate sections before making your selection.

[x] Life sciences [ ] Behavioural & social sciences [ ] Ecological, evolutionary & environmental sciences

For a reference copy of the document with all sections, see nature.com/documents/nr-reporting-summary-flat.pdf

Life sciences study design

All studies must disclose on these points even when the disclosure is negative.

Sample size	Sample sizes used in this study referred to previous studies performed in the same laboratory or by other published studies used similar methods as bellow:<br>1. Shen S, et al. Gut microbiota is critical for the induction of chemotherapy-induced pain. Nat Neurosci. 2017 Sep;20(9):1213-1216.<br>2. Chen Q, et al. Dysfunction of cortical GABAergic neurons leads to sensory hyper-reactivity in a Shank3 mouse model of ASD. Nat Neurosci 2020, 23 (4), 520-532.<br>3. Peng M, et al. Battery of behavioral tests in mice to study postoperative delirium. Sci Rep 2016, 6, 29874.<br>4. Li Y, et al. Distinct subnetworks of the thalamic reticular nucleus. Nature. 2020 Jul;583(7818):819-824. doi: 10.1038/s41586-020-2504-5.
Data exclusions	No data was excluded from the analysis
Replication	A minimum of three replicates for all behavioral experiments were performed by blinded investigator with similar results.
Randomization	Mice were randomly allocated into sham or surgery groups for behavioral test and imaging.
Blinding	The investigators for all behavioral tests and analysis are blinded; experimenters performing optogenetics and acquiring fiber photometry images while optogenetic stimulation were not blinded due to single animal group; investigators were blinded for imaging data analysis.

Reporting for specific materials, systems and methods

We require information from authors about some types of materials, experimental systems and methods used in many studies. Here, indicate whether each material, system or method listed is relevant to your study. If you are not sure if a list item applies to your research, read the appropriate section before selecting a response.

Materials & experimental systems

n/a	Involved in the study	
[ ]	[x]	Antibodies
[x]	[ ]	Eukaryotic cell lines
[x]	[ ]	Palaeontology and archaeology
[ ]	[x]	Animals and other organisms
[x]	[ ]	Clinical data
[x]	[ ]	Dual use research of concern
[x]	[ ]	Plants

Methods

n/a	Involved in the study	
[x]	[ ]	ChIP-seq
[x]	[ ]	Flow cytometry
[x]	[ ]	MRI-based neuroimaging

Antibodies

Antibodies used	Rabbit anti-VGLUT, MilliporeSigma, Catlog# G6642<br>Rabbit anti-GABA, Sigma-Aldrich, catlog# A2052<br>Guinea pig anti-TRPV1, Invitrogen, Catlog # A2052<br>Rabbit anti-CB1, Invitrogen, Catlog# PA585080<br>Mouse anti-GFP polyclonal, Invitrogen, Catlog# A6455<br>Mouse anti-Parvalbumin, EMD Millipore Corporation, Catlog# MAB1572<br>Goat anti-rabbit Cy3, Jackson ImmunoResearch,Catlog# 111-165-003<br>Donkey anti-mouse FITC, Jackson ImmunoResearch, Catlog# 715-545-020<br>Goat anti-Guinea pig Cy3, Jackson ImmunoResearch, Catlog#: 106-165-003
Validation	Rabbit anti-VGLUT: https://www.sigmaaldrich.com/US/en/search/g6642?focus=products&page=1&perpage=30&sort=relevance&term=g6642&type=product<br>Rabbit anti-GABA: https://www.sigmaaldrich.com/US/en/product/sigma/a2052?gclid=EAIaIQobChMIkNrjmKnl-wIVl7bICh1Y8w1XEAAYAiAAEgIVr_D_BwE&gclsrc=aw.ds<br>Guinea pig anti-TRPV1: https://www.fishersci.com/shop/products/anti-vr1-polyclonal-pa129770/PIPA129770<br>Rabbit anti-CB1: https://www.fishersci.com/shop/products/cannabinoid-receptor-1-rabbit-anti-human-mouse-rat-polyclonal-invitrogen/PIPA585080?searchHijack=true&searchTerm=PA585080&searchType=RAPID&matchedCatNo=PA585080<br>Mouse anti-GFP polyclonal: https://www.fishersci.com/shop/products/molecular-probes-gfp-rabbit-serum-polyclonal-antibody/A6455#?keyword=A6455<br>Mouse anti-Parvalbumin: https://www.emdmillipore.com/US/en/product/Anti-Parvalbumin-Antibody,MM_NF-MAB1572<br>Goat anti-rabbit Cy3: https://www.jacksonimmuno.com/catalog/products/111-165-003<br>Donkey anti-mouse FITC: https://www.jacksonimmuno.com/catalog/products/715-545-020<br>Goat anti-Guinea pig Cy3: https://www.jacksonimmuno.com/catalog/products/106-165-003

Animals and other research organisms

Policy information about studies involving animals; ARRIVE guidelines recommended for reporting animal research, and Sex and Gender in Research

Laboratory animals	Adult male and female C57/BL6 and PV-Cre knockin (Jax 008069) mice (16-24 weeks old) were purchased from the Jackson Laboratory (ME).
Wild animals	None
Reporting on sex	Animal sex was considered in this study, we used equal number of male and female mice for behavioral assessments. For fiber photometry and EEG/EMG experiment, we didn't see any difference between male and female mice.
Field-collected samples	None
Ethics oversight	All animal use and procedures applied according to protocols approved by the Massachusetts General Hospital Institutional Animal Care and Use Committee (IACUC). Experiments performed were complying with the guidelines established by NIH and the International Association for the Study of Pain.

Note that full information on the approval of the study protocol must also be provided in the manuscript.
